# From campaign to continuity: stakeholders’ recommendations for integrating HPV vaccination into Nigeria’s healthcare system

**DOI:** 10.1186/s12913-026-14225-7

**Published:** 2026-02-18

**Authors:** Catherine Nabiem Akpen, Edikan Uwatt, Mahfus Dauda, Stallone Ngobua, Daradara Kubura, Sidney Sampson, Sunday Atobatele, Adeyinka Ogunsanya, Hilary Okagbue

**Affiliations:** 1 Sydani Institute for Research and Innovation, Sydani Group, Abuja, Nigeria; 2 Sydani Initiative for International Development, Sydani Group, Abuja, Nigeria; 3 Sydani Consulting, Sydani Group, Abuja, Nigeria; 4 Department of Mathematics, Covenant University, Ota, Nigeria

**Keywords:** HPV vaccine, Immunization, Routinization, Vaccination, Healthcare, Thematic analysis

## Abstract

**Background:**

For Human Papillomavirus (HPV) vaccination to be delivered effectively, countries must transition from campaign-based introduction to integration within the routine immunization schedule. While existing literature provides insights into the effectiveness of HPV campaigns and barriers to initial vaccine acceptance, there is a dearth of stakeholder-informed implementation research around the transition from campaign to routine delivery. This study aimed to explore the experiences and perspectives of key stakeholders involved in the HPV vaccine campaign in Nigeria to generate stakeholder-driven recommendations for the effective and sustainable integration of the vaccine into the country’s routine immunization schedule and strengthen national capacity for introducing and sustaining future new vaccines.

**Methods:**

This study adopted a qualitative, exploratory research design. The research was conducted across nine Nigerian states, representing five of the six geopolitical zones. Participants were purposively sampled, encompassing 140 stakeholders (30 Key Informant Interviews, 110 In-depth Interviews). Data were collected using semi-structured interview guides, audio-recorded, transcribed verbatim, and thematically analysed.

**Results:**

Stakeholder recommendations emphasize that effectively integrating the HPV vaccine into routine immunization in Nigeria demands a multi-pronged strategy. This includes comprehensive strategies for awareness, engagement, and mobilization; continuous workforce training and motivation; robust data systems and reporting; effective management of misinformation; and intensification campaign. Additionally, early and thorough planning, fostering government ownership, optimizing operational logistics, ensuring consistent funding, and maintaining vaccine availability are critical. These insights provide valuable, transferable lessons for the introduction and sustained implementation of future vaccines.

**Conclusion:**

The transition of the Human Papillomavirus (HPV) vaccine from a campaign-driven approach to routine immunization in Nigeria represents a critical juncture, offering both significant challenges and unparalleled opportunities to strengthen the national health system. Policy priorities should include institutionalizing periodic intensification programs, strengthening community engagement, standardizing immunization data systems, securing sustainable financing, and enhancing primary healthcare infrastructure. By addressing these priorities, Nigeria can achieve equitable HPV vaccine coverage and strengthen its capacity for future vaccine initiatives, ensuring a lasting public health impact.

**Clinical trial number:**

Not applicable.

**Supplementary Information:**

The online version contains supplementary material available at 10.1186/s12913-026-14225-7.

## Background

Cervical cancer ranks as the fourth most common cancer among women worldwide, with an estimated 604,000 new cases and 342,000 deaths reported in 2020 alone [1]. Nearly 90% of these deaths occur in low and middle-income countries (LMICs), where limited access to screening, treatment, and preventive healthcare services contributes to the high disease burden [2]. The primary cause of cervical cancer is persistent infection with high-risk strains of the human papillomavirus (HPV), particularly types 16 and 18, which are responsible for approximately 70% of all cases [3]. HPV is a widespread sexually transmitted infection, underscoring the urgent need for effective prevention strategies.

The development and rollout of HPV vaccines mark a significant milestone in global public health efforts to combat cervical cancer [4]. These vaccines provide safe and effective protection against the most harmful HPV types, with strong evidence indicating that vaccination, especially when administered before the onset of sexual activity, substantially reduces the risk of HPV infection and subsequent cervical cancer. In light of this, the World Health Organization (WHO) recommends routine HPV vaccination for girls aged 9 to 14 as a foundation for its global initiative to eliminate cervical cancer as a public health threat [5].

As of 2023, more than 130 countries have incorporated HPV vaccines into their national immunization programs. Despite this progress, global coverage remains inconsistent, with many countries, particularly in sub-Saharan Africa, experiencing low uptake rates [6]. Several factors contribute to this disparity, including vaccine supply limitations, hesitancy and misinformation, cultural resistance, and the limited accessibility of adolescent health services [7].

To overcome these challenges and accelerate HPV vaccine coverage, many nations, including Nigeria, have adopted campaign-style delivery approaches. These are typically short-term, high-intensity efforts aimed at vaccinating large numbers of school-aged girls, often with the support of global partners like Gavi, the Vaccine Alliance. While these campaigns have been effective in creating awareness and achieving high initial coverage, they are inherently time-bound and difficult to sustain. Once campaigns conclude, maintaining coverage and ensuring long-term vaccine delivery becomes a significant hurdle, particularly in settings with constrained health resources and competing priorities [8, 9].

For lasting impact, countries must shift from campaign-based rollouts to integrating HPV vaccination into routine immunization (RI) systems, a process known as routinization. This approach involves the systematic and ongoing delivery of the vaccine through existing healthcare infrastructure [6, 10]. Unlike infant immunization programs that rely on fixed facilities, HPV vaccination targets pre-adolescent and adolescent girls, a group with limited routine interaction with health services. Therefore, routinization requires tailored strategies such as school-based outreach, adolescent-friendly clinics, and outreach to out-of-school girls. Achieving this transition demands cross-sector collaboration, ongoing community mobilization, and sustained investments in workforce training, cold chain systems, and health information infrastructure [11, 12].

Despite the importance of routinization, this transition presents complex operational, logistical, and behavioural challenges. Health workers may face increased workloads or insufficient training on adolescent health, and without continued social mobilization, public demand and awareness may decline, policy attention may shift once donor-funded campaigns conclude, and implementation responsibilities may become unclear in decentralized health systems. Furthermore, misinformation and sociocultural concerns, particularly those related to sexuality and reproductive health, continue to fuel vaccine hesitancy in some communities, posing additional barriers to routine uptake [13, 14].

Although existing studies shed light on the effectiveness of HPV vaccine campaigns and the barriers to initial acceptance [15–17], a notable gap remains in implementation research informed by stakeholders. Much of the current literature emphasizes coverage rates and community-level perceptions, offering limited insight into the day-to-day realities experienced by those on the frontlines of service delivery [6, 18]. Yet, the perspectives of key stakeholders, such as health workers, community leaders, and policymakers, are critical. These actors bring valuable, context-specific knowledge of what works, what doesn’t, and what system adaptations are necessary for success. Their input is essential for designing strategies that are both contextually relevant and operationally feasible [19].

In Nigeria, the introduction of the HPV vaccine followed a familiar global pattern: a high-profile campaign targeting girls aged 9–14 years, followed by plans to integrate the vaccine into the routine immunization (RI) schedule. The phase two campaign in nine Nigerian states achieved over 80% coverage. These levels of uptake demonstrated the feasibility of large-scale implementation and routinization. However, sustainability and access remain especially challenging because campaigns are resource-intensive and logistically demanding. This provided a basis for considering routinization as a strategy to ensure continuous coverage. Our research team was actively engaged during the campaign phase, gathering data and collaborating with key stakeholders, health professionals, school officials, caregivers, and community leaders. As Nigeria embarks on the critical phase of routinization, it is important to reflect on early experiences and gather stakeholder insights to inform future planning and policy.

This study aims to explore the experiences and perspectives of key stakeholders involved in the HPV vaccine campaign in Nigeria to generate stakeholder-driven recommendations for the effective and sustainable integration of the vaccine into the country’s routine immunization schedule. By capturing context-specific insights from those directly engaged in planning, delivery, and community mobilization, the study seeks to inform policy and programmatic strategies to strengthen HPV vaccine delivery in low-resource settings and support the long-term success of HPV vaccination efforts in Nigeria.

This effort aligns with the World Health Organization (WHO) cervical cancer elimination goals of reducing cervical cancer incidence to fewer than four cases per 100,000 women [17]. The challenge of sustaining HPV vaccination is not just technical, but also a public health imperative. Capturing and analyzing stakeholder experiences from Nigeria’s HPV campaign rollout generates actionable recommendations to support the effective routinization of the HPV vaccine and to build national capacity for introducing and sustaining future vaccines.

## Methods

### Study design

This study adopted a qualitative, exploratory research design to investigate stakeholders’ experiences and recommendations regarding the transition of the HPV vaccination post-mass vaccination campaign to routinization, ensuring the successful delivery and completion of “Campaign to Continuity” promises. The qualitative approach was chosen for its strength in eliciting rich, contextual data that reflect stakeholders’ experiences and insights. Key informant interviews (KIIs) and In-depth interviews (IDIs) were adopted to capture perspectives at different levels. KIIs were conducted with policymakers and senior health officials to obtain high-level insights, and IDIs were conducted with frontline health workers and community stakeholders to explore personal experiences, operational challenges, and contextual factors in greater depth. The combination of KIIs and IDIs enabled triangulation of information across different stakeholders and provided a more comprehensive understanding of the implementation process.

### Study setting

The study was conducted in nine Nigerian states: Cross River, Edo, Delta (South-South); Kogi, Kwara, Plateau (North-Central); Ekiti (South-West), Ebonyi (South-East), and Katsina (North-West). These states represent where the Sydani Group was contracted to provide technical support during the phase two rollout. Following the introduction of HPV vaccination in 16 states during phase one in 2023, the country expanded the rollout to the remaining 21 states in phase 2. The Gavi Alliance engaged Sydani Group to provide technical assistance at both national and sub-national levels. At the national level, Sydani Group supported all states in collaboration with the National Primary Health Care Development Agency (NPHCDA), focusing on programmatic needs and available resources for the HPV introduction and the integration of the HPV vaccine into the routine immunization schedule. At the sub-national level, Sydani Group was contracted to provide direct technical assistance in nine states; these nine states formed the study setting. These states represent five of the six geopolitical zones in Nigeria, ensuring regional diversity in socio-cultural, economic, and health system contexts. Detailed demographic and health infrastructure profiles were documented for each state to enhance contextual interpretation of the findings.

### Study participants and sampling strategy

Participants were selected using purposive sampling to ensure the inclusion of stakeholders with direct involvement in or knowledge of the HPV vaccine introduction. The sample encompassed stakeholders across multiple levels of implementation, national, state, local government area (LGA), and ward, as well as civil society organization (CSO) representatives. At the national level, three key officers involved in the HPV vaccine campaign were interviewed. At the state level, participants included high-ranking officials from the State Primary Health Care Board or Agency, LGA-level officers, ward-level officers, and CSO representatives in each state.

The targeted sample size was 140 participants: 30 for Key Informant Interviews (KIIs) and 110 for In-Depth Interviews (IDIs). This composition was designed to ensure comprehensive coverage of all relevant implementation levels and stakeholder groups. While this target served as a planning guide, the actual data collection was informed by the principle of data saturation. All 140 planned participants were interviewed, as they had been identified and informed in advance; their perspectives were considered important for inclusiveness.

### Inclusion and exclusion criteria

Inclusion criteria required participants to have played an active role in the HPV vaccination campaign. Participants were included if they provided informed consent and were available during the study period. Individuals who lacked direct involvement or could not be reached during data collection were excluded.

### Data collection instruments and procedures

Data were collected using semi-structured interview guides tailored to the specific respondent categories. KII guides (Supplementary Material 1) were used for national and state-level stakeholders, while IDI guides (Supplementary Material 2) were used for LGA officers, ward-level health workers, and CSO representatives. The interview guides were developed by the research team in line with the study objectives and a review of relevant literature on immunization program implementation.

Interview questions explored multiple thematic areas: planning and coordination, stakeholder engagement, service delivery, health workforce capacity, supply chain management, data use, supervision, community engagement, and factors influencing acceptance or resistance to the HPV vaccine. Lessons learned, innovative strategies, contextual barriers, and recommendations for a successful transition from mass campaign to routinization were also explored. Interviews were conducted by staff of Sydani Institute for Research and Innovation (SIRI), and participants were interviewed at their offices or other convenient locations. Before each session, verbal informed consent was obtained. All interviews were audio-recorded with permission and transcribed verbatim manually by trained transcribers. To ensure accuracy, each transcript was reviewed by the research team before analysis. The data collection spanned two weeks in November 2024, after the completion of the HPV vaccination campaign, ensuring that participants could reflect on their full experiences and immediate outcomes.

### Instrument validity and reliability

Face and content validity of the interview guides were established through expert review by public health and statistics professionals at SIRI. The instruments were pre-tested with three national-level stakeholders previously involved in HPV vaccine implementation in October 2024. Feedback from the pre-test led to the refinement of ambiguous or irrelevant questions. The pre-test transcripts were analysed using thematic coding to assess the instruments’ clarity, relevance, and ability to elicit meaningful data.

### Theoretical framework and analysis approach

Coding was done using Dedoose while the study employed the Consolidated Framework for Implementation Research (CFIR) [20] alongside Braun and Clarke’s Six-phased method of thematic analysis [21] to explore stakeholders’ insights from Nigeria’s initial HPV vaccine campaign experiences. The aim was to generate actionable recommendations to support the effective routinization of the HPV vaccine and strengthen national capacity for introducing and sustaining future new vaccines. CFIR was selected for its comprehensive approach to identifying and analysing multilevel factors that influence the implementation and sustainability of health interventions. It provided a structured lens for examining key domains such as Intervention Characteristics, Outer Setting, Inner Setting, Characteristics of Individuals, and Process. Data analysis followed Braun and Clarke’s systematic six-phase method: (1) Familiarization with the data through repeated reading of transcripts, (2) Initial coding using both deductive (based on CFIR constructs) and inductive approaches to identify emerging insights, (3) Theme development by organizing codes into meaningful patterns, (4) Reviewing themes to ensure coherence with CFIR domains while preserving participants’ perspectives, (5) Defining and naming themes to clearly articulate each theme’s focus in relation to CFIR constructs, and (6) Reporting of findings to highlight context-specific recommendations for scaling and sustaining the HPV vaccine in routine immunization efforts.

### Reflexivity

Reflexivity was integrated throughout the research process. The multidisciplinary research team, which included public health experts, qualitative researchers, and independent reviewers, maintained reflexive journals to document assumptions, field experiences, and analytical reflections. Regular team debriefs allowed for peer scrutiny and helped mitigate individual biases. Interviewers used neutral, non-leading probes to preserve the authenticity of participant narratives.

### Trustworthiness

To ensure trustworthiness, Lincoln and Guba’s framework [22] was applied. Credibility was enhanced through triangulation of data sources, including interviews, documents, and questionnaires, as well as member checking where feasible. Transferability was supported by providing detailed descriptions of the study settings and participant contexts. Dependability was reinforced by maintaining clear audit trails of the research process. Confirmability was achieved through the documentation of reflexive insights and analytic decisions, along with peer validation of coding outputs.

## Results

### Sociodemographic analysis of the participants

A total of 140 participants, as presented in Table 1, were engaged across the nine states and at the national level, reflecting a diverse mix of gender, age, professional experience, and stakeholder roles relevant to immunization services. The gender distribution showed a slight predominance of female participants, who made up 54% (*n* = 75) of the sample, while males accounted for 46% (*n* = 65). Participants were primarily middle-aged, with nearly half (49%, *n* = 68) between the ages of 41 and 50 years. Fewer participants were under 40 years of age, with only 2% (*n* = 3) between 20 and 30 years, and 13% (*n* = 19) between 31 and 40 years. This reflects a workforce largely composed of experienced professionals.

In terms of educational background, most of the participants held at least a bachelor’s degree (55%, *n* = 77), while 31% (*n* = 43) had postgraduate qualifications. A smaller proportion (14%, *n* = 20) possessed a diploma. Participants with higher academic qualifications were spread across several states, suggesting broad professional development within the immunization workforce.

Years of experience in current roles varied. Almost half (45%, *n* = 63) had worked in their roles for between 2 and 5 years, while 26% (*n* = 36) had served for 6–10 years. A significant number (23%, *n* = 32) had been in their positions for over a decade, and a small proportion (6%, *n* = 9) were relatively new to their roles, with less than one year of experience.

Participants represented a wide array of stakeholder groups involved in immunization programs. These included Immunization Officers (23%, *n* = 32), Ward Focal Persons (21%, *n* = 29), and Health Promotion Officers (11%, *n* = 16). Others included Civil Society Organization representatives (13%, *n* = 18), Monitoring and Evaluation Officers (10%, *n* = 14), Cold Chain Officers (8%, *n* = 12), State Mobilization Officers (5%, *n* = 7), and senior-level personnel such as executive secretaries, directors, and program managers (9%, *n* = 12). This diversity of roles enriched the perspectives shared during the study and provided a broad understanding of immunization system experiences across levels and contexts.

**Table 1:** Socio-demographic characteristics of participants

Characteristic	Cross river	Delta	Edo	Ebonyi	Ekiti	Katsina	Kwara	Kogi	Plateau	National	Total, *n* (%)
**Gender**											
Male	5	4	4	13	3	15	4	5	10	2	65 (46)
Female	10	12	11	2	14	1	8	10	6	1	75 (54)
**Age Group**											
20–30	1	0	0	1	0	0	0	0	1	0	3 (2)
31–40	3	1	2	1	4	1	2	4	1	0	19 (13)
41–50	5	4	10	8	8	8	7	7	8	3	68 (49)
51–60	6	11	3	5	5	7	3	4	6	0	50 (36)
**Education**											
Diploma	1	1	1	1	1	8	0	2	5	0	20 (14)
Bachelor’s degree	7	8	10	9	7	7	9	11	9	0	77(55)
Postgraduate	7	7	4	5	9	1	3	2	2	3	43 (31)
**Years in Current Role**											
< 1 year	4	1	0	0	3	0	0	0	1	0	9 (6)
2–5 years	6	8	8	4	9	4	6	5	11	2	63 (45)
6–10 years	2	5	3	2	4	4	4	9	3	0	36 (26)
> 10 years	3	2	4	9	1	8	2	1	1	1	32 (23)
**Stakeholder Group**											
Immunization Officers	3	2	3	3	5	4	4	2	6		32 (23)
Cold Chain Officers	1	1	1	0	1	2	3	2	1		12 (8)
Ward Focal Person	4	4	4	3	2	4	2	3	3		29 (21)
Civil Society Organizations	2	2	2	2	2	2	2	2	2		18 (13)
State mobilization officers	0	0	0	0	0	2	1	3	1		7 (5)
Monitoring and Evaluation Officers	3	1	2	4	2	0	0	2	0		14 (10)
Health Promotion Officers	0	4	2	2	5	2	0	0	1		16 (11)
Others (Executive secretaries, directors, managers)	2	2	1	1	0	0	0	1	2	3	12 (9)

### Thematic analysis and synthesis

Thematic analysis and synthesis of stakeholder interviews reveal critical actions required to effectively and sustainably integrate the HPV vaccine into Nigeria’s routine immunization schedule. The key findings are summarized in Table 2.

**Table 2:** A thematic summary of key findings from the study

S/*N*	Themes	Description / Participant Insights
1	Comprehensive Communication and Engagement	Respondents emphasized that successful HPV vaccine integration requires continuous communication, strong community engagement, and active mobilization. They highlighted the need for consistent advocacy, use of local languages, schools as access points, and collaboration with trusted stakeholders and sectors like education. They warned that misinformation remains a major barrier and recommended engaging credible figures and platforms to build confidence and counter vaccine hesitancy.
2	Continuous Training and Workforce Motivation	Respondents emphasized that a well-trained, motivated, and adequately staffed healthcare workforce is central to successful HPV vaccine integration. They highlighted the need for continuous training, capacity building, and emphasis on permanent integration into routine immunization, alongside addressing challenges like understaffing and low motivation to ensure sustained coverage and vaccine acceptance.
3	Strengthening Data Systems and Reporting	Respondents noted that weak data management driven by limited training, outdated tools, and poor use of systems like DHIS2 undermines HPV vaccination programs. They recommended continuous staff capacity-building, updated standardized tools, stronger accountability systems, and sensitization of health workers to ensure accurate and consistent data capture.
4	Intensification campaign	Respondents noted that sustaining HPV vaccination after mass campaigns is a global challenge, as shifting to facility-based delivery creates access barriers. They recommended intensification campaigns in schools and communities to maintain demand and uptake.
5	Fostering Ownership	Respondents emphasized that ownership is vital for HPV vaccination success, with both state and national governments expected to provide financial and implementation support. They noted that reliance on external partners is unsustainable and stressed the need for shared responsibility to ensure continuity and effectiveness.
6	Early and Comprehensive Planning	Respondents stressed that successful HPV vaccine integration depends on early and comprehensive planning to prevent oversights, ensure stakeholder engagement, and manage competing priorities. They highlighted robust supply chains and timely funding as critical pillars, noting that when aligned within structured planning, these elements strengthen delivery, build community trust, and support long-term sustainability.

1. Comprehensive Communication and Engagement

Respondents emphasized that the successful integration of the HPV vaccine into routine immunization programs requires a holistic and continuous approach to communication, community engagement, and mobilization. They noted that ongoing awareness campaigns are vital for informing the public about the vaccine’s benefits, dispelling myths, and reinforcing its place within routine immunization. Respondents further stressed the importance of securing community buy-in through consistent advocacy and sensitization, particularly by engaging trusted stakeholders such as parents, traditional and religious leaders, influencers, and Civil Society Organizations (CSOs). They explained that delivering messages in local dialects, using multiple communication platforms, and leveraging schools as key access points are practical strategies for effectively reaching eligible populations.

In addition, respondents underscored the importance of intersectoral collaboration and alignment, especially with the Ministry of Education and other partner organizations, to strengthen school-based delivery and ensure coherence from national to local levels. At the same time, they warned that misinformation, particularly through social media and influential individuals, remains a major barrier. To address this, respondents recommended proactively engaging trusted figures and credible platforms to amplify accurate information, build confidence, and overcome vaccine hesitancy.

*Sensitization period should be extensive before the actual rollout. This allows people to be fully informed about the benefits and the target groups. Efforts should include convincing community leaders*,* chiefs*,* and other influential figures*,* as they can disseminate the information effectively within their communities. Limited sensitization often results in low turnout and uptake*,* so adequate preparation and engagement are critical to success*. **IDI_ESKT_Ekiti SW LG_Igbaraodo Ward_**

*The use of vaccine champions*,* who are members of the community*,* was very effective. If individuals from outside the community had come to share the information*,* it would have been difficult for the people to accept it. However*,* because the vaccine champions were known*,* respected*,* and trusted members of the community*,* the information was readily accepted. In fact*,* the community members were even more enthusiastic. I believe that if we had been the ones conducting the sensitization*,* the response might not have been as positive. For any future vaccine introduction*,* I strongly recommend adopting this approach.***(KII_PL_CSO2)**

*As I mentioned earlier*,*… the focus of sensitization should be directed more toward schools and churches. These platforms can help spread the message and address resistance from those areas. However*,* the primary emphasis should remain on schools since the target group will primarily be reached there. ***(KII_DE_IkaNorthEast)**

*During the HPV campaign*,* social media played a significant role in undermining the program. Instead of using the platform to promote the benefits of the HPV vaccine*,* misinformation was widely spread*,* claiming the vaccine was unsafe….For future campaigns*,* there should be efforts to regulate misinformation on social media*,* ensuring that individuals do not spread false claims about topics they are not well-informed on… Involving well-known individuals or platforms to openly endorse the vaccine could significantly influence public opinion… People tend to trust those at the top more than field workers*,* so having these influencers vouch for the vaccine would carry more weight and credibility*. **(IDI_KOG_OGORI MAGONGO)**

2. Continuous Training and Workforce Motivation

The successful integration of the HPV vaccine into routine immunization programs depends heavily on having a well-trained, knowledgeable, and motivated healthcare workforce. Respondents emphasized that continuous training and strong capacity building are essential and not optional for ensuring sustained success and equitable access to the vaccine.

They explained that effective vaccine delivery requires comprehensive and ongoing training programs to equip health workers with the necessary skills for vaccine administration, cold chain management, and community education. They mentioned that these trainings should also stress the permanent integration of HPV vaccination into routine immunization schedules, supporting a long-term, sustainable approach.

Beyond training, respondents highlighted the need to address workforce challenges such as understaffing, which can hinder consistent follow-up in schools and other target areas. Finally, they stressed that improving health worker motivation and assigning dedicated personnel are crucial strategies to strengthen vaccine acceptance and achieve broad coverage.

*…if health workers are motivated*,* they can create time to visit schools*,* gather the girl children*,* and talk to them about HPV. Motivated health workers will make time for this. As the saying goes*,* ‘money stops nonsense’—with adequate funding*,* people will work effectively and provide results*,* but without it*,* they may be reluctant to go out and engage. ***(IDI_CRS_Calabar_muncipal)**

*My recommendation is that health workers should be trained and equipped with the knowledge they need to effectively impact the people they serve.* (**IDI_Ikot-Effanfa)**

*Additionally*,* we have health facility training*,* and it should not be discontinued. The training we had was specifically for the campaign*,* which included the introduction of the vaccine into the immunization schedule. However*,* some people believe that the training should end with the campaign*,* but it is an ongoing process. More orientation is needed at both the state and ward levels to emphasize that this vaccine is now part of the routine immunization schedule and is here to stay. ****(*IDI_KW_Offa)**

3. Strengthening Data Systems and Reporting

Respondents highlighted that weak data management and reporting are major challenges to effective HPV vaccination programs. They explained that these issues stem largely from insufficient training and outdated tools. According to respondents, many health workers and Monitoring and Evaluation (M&E) officers lack adequate skills in using digital health information systems such as DHIS2, which often results in underreporting and inconsistencies between the number of vaccines distributed and those officially recorded as administered.

Respondents further noted that the reliance on outdated and non-standardized data collection tools compounds the problem, as these templates cannot accurately capture information for newer vaccines like HPV. To address these gaps, they recommended multi-faceted interventions, including continuous capacity-building for staff, the use of updated and standardized data collection tools, and the establishment of stronger accountability systems. They also emphasized the importance of sensitizing health workers on the critical role of accurate and consistent HPV data capture at every stage of the program.

*We have that knowledge gap for the data team to try to key in this data into DHIS 2. So*,* you can have more like refresher training*,* yes*,* for the data team so that they will know how to key in this because data is the key. Whatever you’re doing is not documented*,* you’ve not done it. ***(KII_state-Level)**

*We noticed that the facilities actually had HPV and utilized it. But the problem was with the M&E. So the M&E did not know where exactly to enter HPV until we did a one-on-one mentoring and coaching… Now all the LGAs are actually reporting.* **(KII_CRS)**

*Even the last time I went to Abuja*,* they gave me the number of children we vaccinated during this immunization. I was all looking at myself because I know the number of vaccines we have issued out… But what they showed me there is that we have vaccinated only 5*,*000. ***(KII_state-Level)**

*Well*,* I think health workers need to be sensitized*,* and there is also a need to address the issue with data tools. This has been a common complaint from some of our technical partners*,* especially Sydani*,* as most of our data tools do not capture HPV*,* and many health workers are not reporting it. If we are routinizing HPV vaccination*,* we need to ensure it is fully integrated. Like other vaccines*,* our data tools should reflect this*,* and health workers should understand that reporting HPV is part of their responsibilities.* (**KII_KW)**

4. Intensification campaign

Respondents highlighted the global challenge of sustaining momentum in HPV vaccination after mass campaigns, noting that this issue is not unique to Nigeria. They explained that the shift from active, campaign-style delivery to facility-based vaccination often creates barriers, since individuals are now required to visit health centers to access the vaccine. To address this, respondents suggested intensification campaigns targeting schools and communities as a way to maintain demand and improve uptake.

*Regarding the routinization process*,* it’s important to note that this is not unique to Nigeria; globally*,* the momentum for HPV vaccine uptake tends to decline after the MAC campaign. However*,* with increased sensitization efforts*,* the goal can still be achieved. Currently*,* there are no teams actively going out*,* so girls have to visit health facilities to receive the vaccine. The question then becomes: how do we encourage people to visit health facilities*,* or how do we take the vaccine to those who need it? One approach could be organizing an annual intensification program for HPV*,* as many countries are doing. This could involve targeting schools specifically for HPV vaccination.* (**KII with National)**

5. Fostering Ownership

Respondents emphasized that ownership is a critical factor for the success of HPV vaccination as part of routine immunization. They noted that both state and national governments need to contribute financially and take an active role in program implementation. According to respondents, relying solely on external partners is not sustainable for long-term success. Instead, they suggested that the program should be framed as a shared responsibility, with collective efforts seen as essential for ensuring continuity and effectiveness over time.

*The government*,* whether at the state level in Ekiti or at the national level in Nigeria*,* needs to contribute in one way or another. We cannot simply sit back and expect everything to be provided for us. In which areas are we actively participating? It is only when we see the program as our own and take ownership that it will yield better outcomes. (***IDI_EKST_Ido LG_IIfakia)**

6. Early and Comprehensive Planning

Respondents emphasized that the successful integration and sustained delivery of HPV vaccines rely on early, comprehensive, and well-structured planning. They explained that this proactive approach helps to avoid critical oversight, ensures community engagement, and secures the active participation of key stakeholders, particularly at sub-national levels. According to respondents, rushed or fragmented planning often results in logistical failures, weak community buy-in, and missed opportunities to reach underserved populations.

They further highlighted that in resource-constrained primary healthcare settings, competing priorities can undermine program execution, making structured planning essential to minimize conflicts and maintain focus on HPV vaccination goals.

In addition, respondents identified two interconnected pillars of program success: a robust supply chain and timely funding. They noted that strong supply chains ensure vaccines reach the right places at the right time, while adequate and timely funding enables operational efficiency, outreach, and mobilization of health teams. Respondents stressed that when these elements are aligned within a well-planned framework, they not only strengthen program delivery but also build community trust and acceptance, ultimately driving higher coverage and long-term sustainability.

*This routine requires adequate funding. We need funds to conduct outreach services because without money*,* it becomes impossible to transport materials or carry out activities. ***(IDI_KOG_Ogori Magongo)**

*The recommendation is for management to improve forecasting. Proper forecasting is essential because if caregivers arrive at a service point only to be turned away due to a lack of supplies*,* they may not be able to return. This could negatively impact the plan and overall coverage. Ensuring the availability of adequate supplies*,* especially vaccines and management tools*,* at all times is crucial to maintaining service delivery without disruption. ***(KII_KT_SIO)**

*For future exercises*,* my recommendation is that at both the national and state levels*,* planning should be well-coordinated and time-bound. This is important because*,* at certain stages*,* there was insufficient time for engagement and other preparatory activities before implementation. Additionally*,* there were numerous competing priorities*,* particularly within the primary healthcare department. ***(IDI_PL_Bakin Ladi LG)**

*A number of recommendations*,* including the need for thorough planning*,* ensuring the inclusion of all stakeholders*,* particularly at the sub-national level*,* and implementing comprehensive community engagement activities. Additionally*,* developing a clear crisis communication plan and identifying spokespersons*,* especially at the sub-national level*,* to address any vaccine-related crisis is needed.* (**KII Director ACSM )**

## Discussion

Findings from our study highlight that successful HPV vaccine routinization in Nigeria hinges on a synergy of Comprehensive Communication and Engagement, Continuous Training and Workforce Motivation, Strengthening Data Systems and Reporting, Intensification campaign, Fostering Ownership, and Early and Comprehensive Planning.

This study explored the experiences and perspectives of key stakeholders involved in the HPV vaccine campaign in Nigeria to generate stakeholder-driven recommendations for the effective and sustainable integration of the vaccine into the country’s routine immunization schedule. The findings offer diverse state and national perspectives to inform strategies that not only ensure the effective routinization of the HPV vaccine but also strengthen the country’s capacity to introduce and sustain future vaccines.

Our study showed that initial HPV vaccination experience revealed low public awareness and the proliferation of false narratives, impeding vaccine acceptance, which is similar to findings from previous studies [23, 24]. This highlights the need to move beyond simple information sharing toward a more comprehensive approach that combines awareness, engagement, and mobilization. Continuous sensitization is essential to dispel myths and emphasize the benefits of the vaccine. To achieve this, efforts should focus on strengthening community buy-in by involving parents, traditional rulers, religious leaders, and influencers. At the same time, intersectoral collaboration and stakeholder alignment must be fostered through meaningful partnerships and shared understanding, particularly with key ministries such as the Ministry of Education and other relevant organizations at all levels. Comparatively, countries such as Rwanda and Senegal have successfully transitioned HPV vaccines into routine immunization, with Rwanda achieving a remarkable 93.23% coverage by implementing a public-private community partnership, school-based vaccination model in collaboration with the Ministry of Education, fostering active community involvement [25]. Senegal’s success was driven by multi-sectoral collaboration and engagement with key stakeholders. The country’s sustainability approach emphasizes enhancing stakeholder understanding and strengthening communication strategies to ensure long-term program success [26].

Several studies agree with the findings of our study, which show that successful integration of the HPV vaccine into routine immunization programs relies on a well-trained, motivated, and adequately staffed healthcare workforce [27–29]. This calls for continuous training programs to equip health workers with the technical skills needed for vaccine delivery, cold chain management, and community education. These efforts must also address workforce attrition, tackle understaffing to enable consistent follow-up in schools and other target areas, and enhance health worker motivation. Analysis of responses from our study shows that previous HPV campaigns face challenges with data management, like insufficient training on digital health information systems (DHIS2) and reliance on outdated tools, which lead to significant underreporting and data discrepancies, as seen in other studies [30]. To address this, there is a need to strengthen data systems, standardize tools, and provide continuous training and mentorship for data teams, which would result in a ripple effect of consistent data integrity, accountability, and informed strategic planning. Australia has implemented a deliberately designed holistic data system that captures linked data on HPV vaccination rates and outcomes. This approach has proven particularly effective in assessing vaccine effectiveness, highlighting the benefits of a strengthened data system [31].

In addition, findings from our study show that previous HPV vaccination campaigns suffered challenges around operational logistics, vaccine availability, and sufficient, timely funding, aligning with other studies [32–34]. Challenges in any of these areas create significant bottlenecks, undermining program objectives and eroding community trust, which could be addressed by strong logistics and a reliable supply chain that guarantees the vaccine’s presence where and when it’s needed. Also, a proactive approach of early and comprehensive planning prevents logistical breakdowns, reduces competing priorities for vaccination in resource-constrained settings, and drastically reduces missed opportunities for HPV vaccination.

Our findings indicate that the long-term success and sustainability of the HPV vaccine program depend on strong government ownership and financial commitment, an observation consistent with previous studies [35, 36]. This calls for the need to move beyond the sole reliance on external partners and implies that the HPV vaccine, like other routine immunizations, must be fully internalized as a national priority with dedicated resources and strong oversight. China provides a compelling example of this approach; the National Health Commission encouraged well-funded local governments to initiate HPV vaccination pilots at the city or provincial level. By the end of 2022, these pilot cities, supported by free or subsidized vaccination policies, achieved coverage rates of 80% or higher among targeted girls. This success can be attributed to strong local government implementation and financial backing [37]. Similarly, Rwanda exemplifies how prioritizing cervical cancer prevention through strong political will, multilevel accountability, and effective resource utilization can drive the successful implementation of HPV vaccination programs [38, 39]. These cases underscore the transformative potential of proactive government engagement and strategic resource allocation. In addition to counteracting the common post-campaign decline in momentum, there is a need to institutionalize intensification campaigns to maintain coverage and consistently reach new eligible cohorts. The importance of such programs is underlined by Burkina Faso’s experience, where 144,000 girls were vaccinated during a week-long intensification campaign in 2023, significantly boosting coverage. Conversely, in Cameroon, the absence of an intensification period in 2024 led to a drop in coverage, highlighting the challenges routine immunization systems face in sustaining high vaccination rates without periodic reinforcements [40].

Recommendations from Nigeria’s initial HPV vaccine campaign from our study offer vital, generalizable lessons for the successful introduction and sustained delivery of any future new vaccine. A comprehensive approach to awareness, engagement, and mobilization is not merely supplementary but a core element for building community trust and continuously addressing misinformation [41]. New vaccine introductions expose underlying weaknesses in the health system, especially in low-resource settings [42], underscoring the critical need to invest in effective logistics and supply chain systems, a well-trained, motivated health workforce, thereby strengthening the entire immunization ecosystem. Strengthening data systems is indispensable, as there is a need to collect, analyze, and utilize accurate, timely data for effective planning, monitoring, and adaptation of any new vaccine program. Lastly, the sustainability of vaccine programs requires strong political commitment, a sense of ownership, and consistent domestic financial investment [43]. This demands a necessary shift from dependence on short-term external support to a more self-reliant and enduring approach.

### Strengths and limitations

The strength of this study lies in its comprehensive thematic analysis of diverse stakeholder insights from multiple states across Nigeria, providing a rich, multi-faceted understanding of the HPV campaign experiences. This qualitative depth offers practical and context-specific guidance.

A limitation is that the insights are perceptions of stakeholders and not direct observational data, which might introduce some subjectivity or recall bias.

### Implications for practice and policy recommendations

**Fig. 1:**
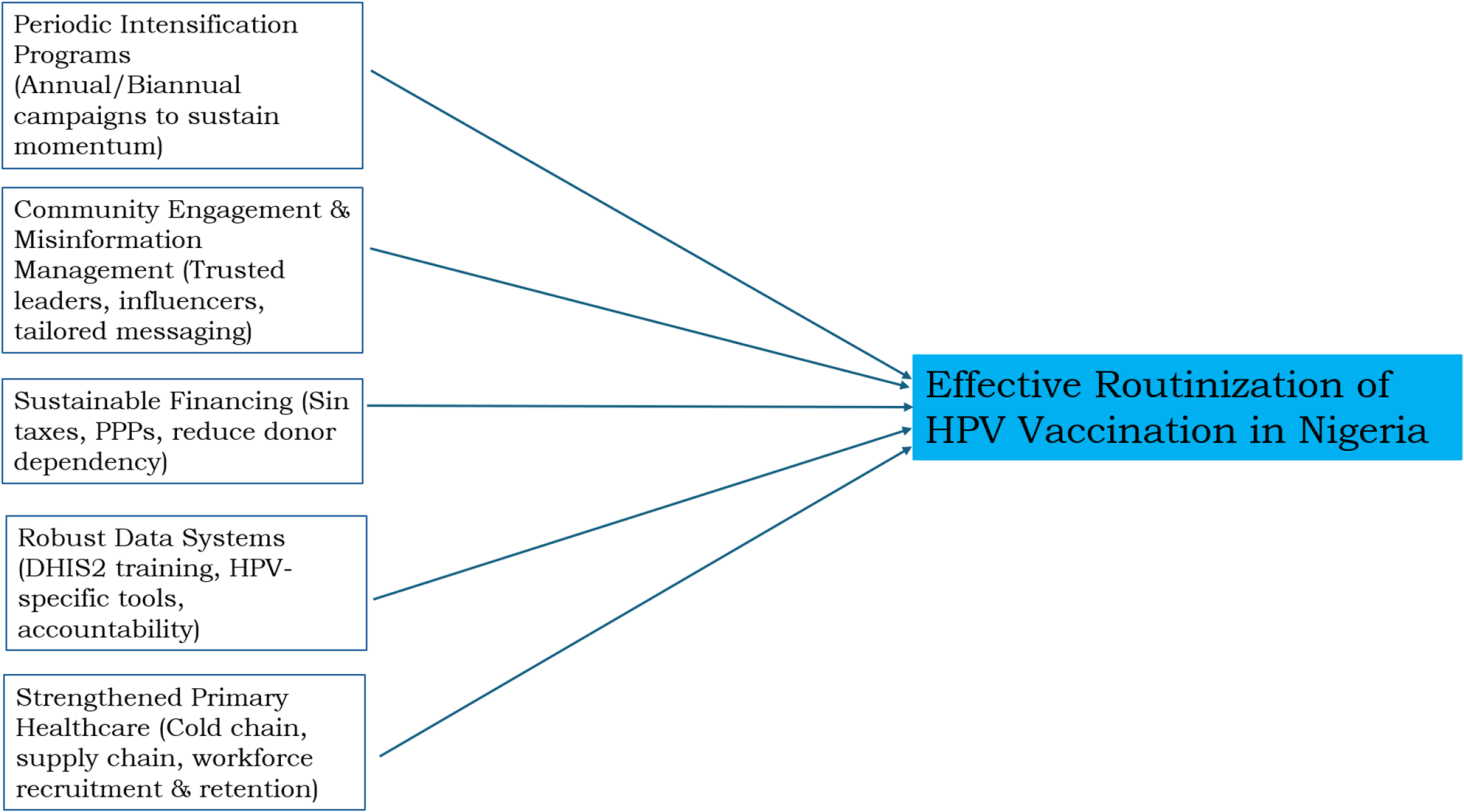
Showing policy and practice recommendations for effective routinization of HPV vaccination in Nigeria

The findings of this study highlight critical considerations for the successful transition of HPV vaccination from campaign mode to routine immunization in Nigeria. Drawing on stakeholders’ perspectives, we propose the following public health practice and policy recommendations as presented in Fig. 1.

First, to sustain momentum and close existing coverage gaps, Nigeria should institutionalize periodic intensification programs for HPV vaccination. These programs, conducted annually or biannually, would complement ongoing routine immunization and campaign-based efforts, especially in hard-to-reach or underserved communities. By creating a predictable and recurring schedule, such initiatives would help ensure consistent access to vaccines and reinforce public awareness over time.

Second, community engagement must remain a central pillar of HPV vaccination strategies, especially in the post-campaign phase. Expanding and tailoring existing community engagement strategies to address HPV-specific concerns can strengthen local advocacy. Involving trusted community leaders and influencers will be instrumental in combating vaccine hesitancy and misinformation [44]. Moreover, integrating HPV messaging into existing default tracing and follow-up mechanisms can reinforce vaccine uptake and bolster trust within communities.

Third, sustainable financing is vital to reduce over-dependence on donor funding for HPV vaccination programs. Nigeria can explore the allocation of sin tax revenues, such as levies on tobacco and alcohol, to support cervical cancer prevention initiatives, including HPV vaccination. Additionally, establishing and scaling up public-private partnerships (PPPs) can unlock new resources from the private sector to support logistics, outreach campaigns, and supply chain infrastructure, ensuring long-term program viability.

Fourth, strong immunization data systems are critical for effective HPV vaccine program monitoring and accountability. Continuous training for healthcare workers and monitoring and evaluation (M&E) officers on platforms like DHIS2 should be prioritized. Tailored data collection tools specific to HPV vaccination must be adopted, and mechanisms for reconciling discrepancies between vaccine stocks and administered doses should be institutionalized. Furthermore, periodic sensitization of health workers on the value of precise data capture will enhance the accuracy and credibility of program assessments.

Lastly, strengthening Nigeria’s primary healthcare infrastructure is a foundational requirement for the successful delivery of immunization services. This includes investing in reliable cold chain and supply chain systems to maintain vaccine integrity from storage to administration. Additionally, efforts must focus on the recruitment, capacity building, and retention of healthcare personnel. A well-trained and adequately supported workforce is indispensable for maintaining high coverage and ensuring equitable vaccine delivery across the country.

## Conclusion

The transition of the HPV vaccine from a campaign-driven approach to routine immunization in Nigeria represents a critical juncture, offering both significant challenges and unparalleled opportunities to strengthen the national health system. The successful transition of HPV vaccination to routine immunization in Nigeria depends on strong communication strategies, resilient supply chains, sustainable financing, a well-trained health workforce, and strengthened data systems. Strong government ownership with sustained domestic investment is essential for long-term success. This study highlights policy next steps, including institutionalizing periodic intensification programs, strengthening community engagement, standardizing immunization data systems, securing sustainable financing, and enhancing primary healthcare infrastructure. By addressing these priorities, Nigeria can achieve equitable HPV vaccine coverage and strengthen its capacity for future vaccine initiatives, ensuring a lasting public health impact.

## Supplementary Information

Below is the link to the electronic supplementary material.

Supplementary Material 1: (KII Guide)

Supplementary Material 2: (IDI Guide)

## Data Availability

The datasets used and/or analyzed during the current study are available from the corresponding author upon reasonable request. However, the interview guides are available as supplementary material.

## Abbreviations

CFIR: Consolidated Framework for Implementation Research
CSO: Civil Society Organization
DHIS2: District Health Information Software 2
Gavi: Gavi, the Vaccine Alliance
HPV: Human Papillomavirus
IDI: In–Depth Interview
KII: Key Informant Interview
LGA: Local Government Area
LMICs: Low–and Middle–Income Countries
M&E: Monitoring and Evaluation
NHREC: National Health Research Ethics Committee
PPPs: Public–Private Partnerships
RI: Routine Immunization
SIRI: Sydani Institute for Research and Innovation
WHO: World Health Organization
