# Supplementary material for: From campaign to continuity: stakeholders’ recommendations for integrating HPV vaccination into Nigeria’s healthcare system
Source: BMC Health Serv Res. 2026 Feb 18;26:394. doi: 10.1186/s12913-026-14225-7 (PMC13020298; doi:10.1186/s12913-026-14225-7)
Supplement: Supplementary file 1 — Supplementary Material 1: (KII Guide) [file 12913_2026_14225_MOESM1_ESM.docx]

**KII GUIDE**

**(National & State Health Officers, Partners in The State)**

**Informed Consent (Oral)**

Good day Sir/Ma, my name is _____________________ and I work for Sydani Group. My organization is currently undertaking a study titled “**HPV Vaccine Introduction: Lessons Learned and Future Directions from the Vaccination Intervention in Nine (9) Nigerian States”**. The study seeks to document and analyze the implementation strategies, achievements, challenges and lessons learned from the HPVVI Phase II project, and to proffer recommendations that could be used to improve prospective vaccine introductions and inform policymaking. I would appreciate it if you could spare some of your time to answer some questions. I assure you that all information shared with me shall be kept in utmost confidentiality. Although the interview is voluntary and you have permission to exit at any time, I would appreciate it if you could complete the interview. Please note that this interview session will be recorded to document what is being discussed adequately.

Do I have your permission to go ahead with the interview? Yes/No

*(End the interview if no, and continue if yes)*

**SECTION A: Socio-demographics**

1. Please, introduce yourself.

Focus: *Prompt where the participant skips any of the following*

- Gender
- Level of educational attainments
- Age
- Designation
- Number of years serving in that designation

**SECTION B: Strategies deployed during the HPV Vaccine Introduction**

1. **Planning and Coordination**

- What is your understanding of the Technical Working Group (TWG)?
- Who were the members of the TWG?
- What were the responsibilities of members of the TWG? (**Probe**: *if all the members have the same responsibilities or members have different responsibilities*)
- How often were the TWG meetings held?
- What were the activities and/or the kinds of discussion that took place at the TWG meetings? (**Probe**: *list of the activities and detailed explanation of each one*)
- What role(s) did the TWG meetings plays in the state’s readiness for the vaccine introduction?
- Who were the members of the expanded Technical Working Group (eTWG)?
- What were the responsibilities of members of the eTWG? (**Probe**: *if all the members have the same responsibilities or members have different responsibilities*)
- What is your understanding of microplanning, as it relates to the vaccine introduction?
- What was the importance of microplanning, as it relates to the vaccine introduction?
- How was microplan development conducted? (**Probe**: *The processes of microplan development, from beginning to end*)
- Who were the officers or people responsible for microplan development?
- How were the developed microplans evaluated and validated? (**Probe**: The validation process, the persons responsible)

1. **Stakeholder Engagement/ACSM**

- Who were the relevant stakeholders identified for the vaccine introduction? (**Prompt**: *Both EPI and nEPI stakeholders*)
- How were the relevant stakeholders identified?
- How were the identified relevant stakeholders engaged?
- What were the contributions of the identified stakeholders to the vaccine introduction?
- How did the involvement and engagement of the stakeholders impact the vaccine introduction?
- How was the vaccine introduction promoted across the state? (**Probe**: *Specific activities, channels used to promote the vaccine introduction across all levels, people and locations visited for promotion*)
- Who were the drivers of promotional activities for the vaccine introduction?
- What roles did the stakeholders play in the promotion of the vaccine introduction?
- How impactful were the promotional activities on the vaccine introduction?
- How was the effectiveness of the promotional activities measured? (**Probe**: *Approach used to monitor and evaluate the promotional activities*)

1. **Financial Management/Funding**

- What the funds were made available for the vaccine introduction? (**Probe**: *Specific names of the funds*)
- What are the sources of the aforementioned funds? (**Probe**: *Names of the organisations, agencies that provided the funds*)
- What purpose did each fund serve, as related to the vaccine introduction? (**Probe**: *Activities, resources that the funds were used for, the teams or people who received the funds*)
- Who was/were the officer(s) in-charge disbursing and monitoring of the released funds?
- What processes were put in place to ensure appropriate allocation and effective monitoring of the funds?
- What type of compensations were provided to the vaccination teams? (**Probe**: *The frequency of payment, the people paid*)

1. **Supply Chain & Logistics**

- What vaccination equipment were made available for the vaccine introduction? (**Probe**: *An exhaustive list of all materials, equipment, tools, etc.*)
- How were the vaccination equipment procured? (**Probe**: *The procurement process, the officers responsible and involved in the process*)
- How were the vaccination equipment distributed to the relevant quarters? (**Probe**: *The distribution plan, officers involved in the development of distribution plan, distribution process*)
- What were the infrastructures put in place to ensure the security of the vaccination equipment? (**Probe**: *The equipment used to secure and maintain the vaccines at appropriate temperature, how data tools and other equipment were safeguarded*)

1. **Service Delivery and Training**

- Who were the personnel recruited to conduct vaccination of the target population? (**Probe**: *Healthcare workers or not, cadres of members of the vaccination teams, team composition*)
- Were the recruited healthcare workers enough for the vaccine introduction?
- What form of training was conducted for the healthcare workers? (**Probe**: *When the training(s) took place, the levels of training, personnel trained across levels*)
- What strategies were used in delivering the vaccines to the target population? (**Probe**: *The different types of vaccination sessions/strategies*)
- How were the strategies deployed? (**Probe**: *How the sessions were conducted, the locations visited*)
- How did the vaccination team manage the wastes generated during vaccination? (**Probe**: *The waste collation, collection and incineration processes, persons responsible*)

1. **Supervision**

- Were supervisions conducted during the vaccination exercise? (**Probe**: *Nature of supervisions, levels of supervisions*)
- Who were the officers responsible for supervision? (**Probe**: *Cadres and affiliation of the supervisors, composition of the supervision teams, if any*)
- What do the supervisors check or look out for during supervision?
- What was the frequency of supervision?

1. **Data Management**

- How was data collection conducted at the vaccination sites? (**Probe**: *Persons in-charge of data recording, the process, the types of data collected*)
- What were the tools used for data collection at the vaccination sites?
- Kindly explain the reporting flow for the collected data. (**Probe**: *The reporting process from the vaccination teams to the appropriate stations, officers involved in the reporting process*)
- What were the validation processes for the vaccination data? (**Probe**: *Detailed explanation of the processes, officers responsible*)

**SECTION C: Successes recorded during the vaccine introduction**

(Instruction: Participant is expected to discuss the achievements recorded across the following intervention areas)

1. What were the achievements recorded during the vaccine introduction:

- Planning & Coordination (**Probe**: *The achievements attributed to: TWG meetings and members involvement, microplanning*)
- ACSM/Stakeholder Engagement (**Probe**: *Results recorded due to stakeholder engagement & involvement; achievements due to promotional activities*)
- Financial Management (**Probe**: *Achievements recorded due to availability of funds*)
- Supply Chain & Logistics (**Probe**: *Achievements recorded due to availability of vaccination resources*)
- Service Delivery and Training (Probe: *Achievements recorded due to delivery strategies, availability of HCWs*)
- Supervision (**Probe**: *Achievements recorded as a result of supervision*)
- Data Management (**Probe**: *Achievements recorded due proper data collection, timely reporting of data*)

**SECTION D: Challenges and mitigants during the vaccine introduction**

(Instruction: Participant is expected to discuss the challenges encountered, factors responsible, followed immediately by mitigants deployed, across the following intervention areas

1. What were the challenges encountered and mitigants deployed during the vaccine introduction?

- Planning & Coordination (**Probe**: *Challenges faced in organizing TWG meetings, developing microplans, validating microplans*)
- ACSM/Stakeholder Engagement (**Probe**: *Challenges faced while engaging stakeholders, planning & conducting promotional activities*)
- Financial Management (**Probe**: *Challenges faced in securing the various funds, allocation and monitoring of the funds, vaccination teams’ payment*)
- Supply Chain & Logistics (**Probe:** *Challenges in procuring vaccines and other materials for the state, distributing to the LGAs; challenges with the CCEs and other storage facilities*)
- Service Delivery and Training (**Probe**: *Challenges faced during vaccination exercise, with recruitment of HCWs, with conducting training, with waste management*)
- Supervision (**Probe**: *Challenges faced during supervision*)
- Data Management (**Probe**: *Challenges with data collection & reporting, and availability of recording and reporting tools*)

**SECTION E: Lessons learned and innovative practices**

1. What were other innovative strategies adopted or implemented during the HPV vaccine introduction in your state? (Probe: The things done differently that positively impacted the project)
2. What were the lessons learned during the HPV vaccine introduction in your state? (Probe: Major failure, what could have been done better)

**Conclusion**

1. What recommendations do you have for future HPV vaccine introduction and other related vaccine?
2. What recommendations do you have for how to routinize the new vaccine?
