# Supplementary material for: From campaign to continuity: stakeholders’ recommendations for integrating HPV vaccination into Nigeria’s healthcare system
Source: BMC Health Serv Res. 2026 Feb 18;26:394. doi: 10.1186/s12913-026-14225-7 (PMC13020298; doi:10.1186/s12913-026-14225-7)
Supplement: Supplementary file 2 — Supplementary Material 2: (IDI Guide) [file 12913_2026_14225_MOESM2_ESM.docx]

**IDI GUIDE**

**(Local Government and Ward Level Officers in the State)**

**Informed Consent (Oral)**

Good day Sir/Ma, my name is _____________________ and I work for Sydani Group. My organization is currently undertaking a study titled “**HPV Vaccine Introduction: Lessons Learned and Future Directions from the Vaccination Intervention in Nine (9) Nigerian States”**. The study seeks to document and analyze the implementation strategies, achievements, challenges and lessons learned from the HPVVI Phase II project, and to proffer recommendations that could be used to improve prospective vaccine introductions and inform policymaking. I would appreciate it if you could spare some of your time to answer some questions. I assure you that all information shared with me shall be kept in utmost confidentiality. Although the interview is voluntary and you have permission to exit at any time, I would appreciate it if you could complete the interview. Please note that this interview session will be recorded to document what is being discussed adequately.

Do I have your permission to go ahead with the interview? Yes/No

*(End the interview if no, and continue if yes)*

**SECTION A: Socio-demographics**

1. Please, introduce yourself.

Focus: *Prompt where the participant skips any of the following*

- Gender
- Level of educational attainments
- Age
- Designation
- Number of years serving in that designation
- In what capacity did you participate during the vaccine introduction?

**SECTION B: Strategies deployed during the HPV Vaccine Introduction**

1. **Planning and Coordination**

- Who were the members of the expanded Technical Working Group (eTWG)?
- Was anybody from your LGA/ward a member of the eTWG? Who?
- How often were the eTWG meetings held?
- What were the responsibilities of members of the eTWG? (**Probe**: *if all the members have the same responsibilities or members have different responsibilities*)
- What is your understanding of microplanning, as it relates to the vaccine introduction?
- What was the importance of microplanning, as it relates to the vaccine introduction?
- How was microplan development conducted? (**Probe**: *Detailed illustration of the microplan development process, from beginning to end*)
- Who were the officers or people responsible for microplan development in your LGA/ward?
- How were the developed microplans evaluated and validated? (**Probe**: The validation process, the persons responsible)

1. **Stakeholder Engagement/ACSM**

- Who were the relevant stakeholders identified for the vaccine introduction in your LGA/ward? (**Prompt**: *Both EPI and nEPI stakeholders*)
- How were the relevant stakeholders identified?
- How was the vaccine introduction promoted across your LGA (**Probe**: *Specific activities, channels used to promote the vaccine introduction, people and locations visited for promotion*)
- What roles did the stakeholders play in the promotion of the vaccine introduction in your LGA/ward?
- How impactful were the promotional activities on the vaccine introduction in your LGA/ward?

1. **Financial Management/Funding**

- What funds were made available for the vaccine introduction in you LGA/ward? (**Probe**: *Names and types of funds*)
- What were the funds used for? (**Probe**: *Activities, resources that the funds were used for*)
- What funds were provided to the LGA teams?
- What type of compensations were provided to the vaccination teams? (**Probe**: *The frequency of payment, the people paid*)

1. **Supply Chain & Logistics**

- What Cold Chain Equipment (CCE), Data Tools, AEFI kits available during the vaccine introduction?
- Were the CCEs available functioning as expected? (**Probe**: *The CCEs that were functioning well*)
- How were the supplies for the implementation distributed to the wards? (**Probe**: *The distribution plan, officers involved in the development of distribution plan, distribution process*)
- What were the infrastructures put in place to ensure the security of the vaccination equipment? (**Probe**: *The equipment used to secure and maintain the vaccines at appropriate temperature, how data tools and other equipment were safeguarded*)

1. **Service Delivery and Training**

- Who were the personnel recruited to conduct vaccination of the target population in your LGA/ward? (**Probe**: *Healthcare workers or not, cadres of members of the vaccination teams, team composition*)
- Were the recruited healthcare workers (HCWs) enough for the vaccine introduction?
- What form of training was conducted for the healthcare workers? (**Probe**: *When the training(s) took place, the levels of training, personnel trained at the LGA and ward levels*)
- What strategies were used in delivering the vaccines to the target population? (**Probe**: *The different types of vaccination sessions/strategies*)
- How were the strategies deployed? (**Probe**: *How the sessions were conducted, the locations visited*)
- How did the vaccination team manage the wastes generated during vaccination? (**Probe**: *The waste collation, collection and incineration processes, persons responsible*)

1. **Supervision**

- Were supervisions conducted during the vaccination exercise? (**Probe**: *Nature of supervisions, levels of supervisions*)
- Who were the officers responsible for supervision? (**Probe**: *Cadres and affiliation of the supervisors, composition of the supervision teams, if any*)
- What do the supervisors check or look out for during supervision?
- What was the frequency of supervision?

1. **Data Management**

- How was data collection conducted at the vaccination sites? (**Probe**: *Persons in-charge of data recording, the process, the types of data collected*)
- What were the tools used for data collection at the vaccination sites?
- Kindly explain the reporting flow for the collected data. (**Probe**: *The reporting process from the vaccination teams to the appropriate stations, officers involved in the reporting process*)
- What were the validation processes for the vaccination data? (**Probe**: *Detailed explanation of the processes, officers responsible*)

**SECTION C: Successes recorded during the vaccine introduction**

(Instruction: Participant is expected to discuss the achievements recorded across the following intervention areas)

1. What were the achievements recorded during the vaccine introduction:

- Planning & Coordination (**Probe**: *The achievements attributed to microplanning*)
- ACSM/Stakeholder Engagement (**Probe**: *Results recorded due to stakeholder engagement & involvement; achievements due to promotional activities*)
- Financial Management (**Probe**: *Achievements recorded due to availability of funds*)
- Supply Chain & Logistics (**Probe**: *Achievements recorded due to availability of vaccination resources*)
- Service Delivery and Training (Probe: *Achievements recorded due to delivery strategies, availability of HCWs*)
- Supervision (**Probe**: *Achievements recorded as a result of supervision*)
- Data Management (**Probe**: *Achievements recorded due proper data collection, timely reporting of data*)

**SECTION D: Challenges and mitigants during the vaccine introduction**

(Instruction: Participant is expected to discuss the challenges encountered, factors responsible, followed immediately by mitigants deployed, across the following intervention areas)

1. What were the challenges encountered and mitigants deployed during the vaccine introduction?

- Planning & Coordination (**Probe**: *Challenges faced developing microplans, validating microplans*)
- ACSM/Stakeholder Engagement (**Probe**: *Challenges faced while engaging stakeholders, planning & conducting promotional activities*)
- Financial Management (**Probe**: *Challenges faced in securing funds for activities, vaccination teams payment*)
- Supply Chain & Logistics (**Probe:** *Challenges in procuring vaccines and other materials for the LGA, distributing to the wards; challenges with the CCEs and other storage facilities*)
- Service Delivery and Training (**Probe**: *Challenges faced vaccination exercise; with recruitment of HCWs, conducting training, waste management*)
- Supervision (**Probe**: *Challenges faced during supervision*)
- Data Management (**Probe**: *Challenges with data collection & reporting, and availability of recording and reporting tools*)

**SECTION E: Lessons learned and innovative practices**

1. What were the innovative practices done to ensure the success of vaccine introduction in your LGA? (Probe: The things done differently that positively impacted the project)
2. What were the lessons learned during the HPV vaccine introduction in your state? (Probe: Major failure, what could have been done better)

**Conclusion**

1. What recommendations do you have for future vaccine introduction?
2. What recommendations do you have for how to routinize the new vaccine?
